# Impact of ComBat harmonization on MRI radiomic-analysis of nasopharyngeal carcinoma: a dual-center study

**DOI:** 10.3389/fonc.2026.1705567

**Published:** 2026-05-08

**Authors:** Wei Pei, Yunyun Wei, Penghao Lai, Lili Mao, Xia Huang, Danke Su, Hai Liao, Shaolu Lu

**Affiliations:** 1 Department of Radiology, Guangxi Medical University Cancer Hospital, Nanning, China; 2 The First Affiliated Hospital of Zhejiang Chinese Medical University (Zhejiang Provincial Hospital of Chinese Medicine), Hangzhou, China; 3 Department of Radiology, Shunde Hospital of Southern Medical University Affiliated Chencun Hospital, Foshan, China; 4 Department of Radiology, Wuzhou Red Cross Hospital, Zhangzhou, China; 5 Department of Nuclear Medicine, Guangxi Medical University Cancer Hospital, Nanning, China

**Keywords:** ComBat harmonization, magnetic resonance imaging, nasopharyngeal carcinoma, prognosis, radiomics

## Abstract

**Purpose:**

To investigate the impact of ComBat harmonization on the performance of 1.5T/3.0T MRI radiomic features in predicting the overall survival(OS) of patients with locally advanced nasopharyngeal carcinoma(LANPC).

**Methods:**

This dual-center retrospective study included 573 patients with LANPC (435 male and 138 female patients). The 3.0T MRI dataset from Hospital 1 was used as the reference batch (training cohort, n=287), and the 1.5T MRI dataset from Hospital 2 was subjected to ComBat harmonization. The 1.5T MRI dataset before ComBat harmonization served as Validation Cohort 1 (n=286), while the 1.5T MRI dataset after ComBat harmonization was designated as Validation Cohort 2. Radiomics features were extracted from the segmented tumors, and principal component analysis (PCA) was applied to assess the batch effect of radiomics features between the two validation cohorts. A radiomics-clinical prognostic model was developed using radiomic features and other clinical factors using multivariate Cox regression. The concordance index (C-index) was used to evaluate model performance in predicting OS, and Kaplan-Meier survival analysis was conducted to explore the impact of the model on the prognosis of LANPC patients.

**Results:**

PCA without ComBat revealed noticeable differences in the first two principal components between batches, indicating a batch effect or unstable radiomic features. Following ComBat harmonization, the principal components showed more consistency between batches, demonstrating radiomics feature stability between batches. Multivariate Cox regression identified EBV-DNA and platelet count as independent clinical factors, which were integrated with the radiomics score to construct the final prognostic model. The C-indexes of the model for predicting OS in the training, validation 1 and validation 2 cohorts was 0.797, 0.610 and 0.648, respectively. The 5-year OS for the model defined low-risk group was significantly better than that of the high-risk group (P < 0.001).

**Conclusion:**

ComBat harmonization effectively reduced the inter-batch effect of radiomic feature sets across different centers and scanners. While the model maintained favorable discrimination for OS and robust risk-group separation via Kaplan-Meier analysis, the primary benefit of ComBat harmonization was the improved qualitative consistency of risk stratification between multi-center datasets, which may support the clinical application of risk-adapted therapy for LANPC.

## 1 Introduction

Nasopharyngeal carcinoma is a malignant tumor arising in the head and neck region, with a high incidence reported in East and Southeast Asia (1). Due to the insidious nature of early symptoms, more than 75% of patients are diagnosed with locally advanced nasopharyngeal carcinoma (LANPC), which significantly reduces overall survival (OS) (2, 3). Numerous models have been published to improve risk stratification and facilitate the development of personalized medicine in LANPC (4).

Medical imaging offers a noninvasive method for monitoring tumor response and progression during treatment. Advances in radiomics have enabled the transformation of visual medical images into mineable high-dimensional data through high-throughput computation (5). Such data captures critical biological and physiological characteristics of tumors, thereby aiding in a comprehensive assessment of tumor heterogeneity (6). Although multiple single-center studies have demonstrated the feasibility of using radiomics features as prognostic biomarkers for NPC, multi-institutional studies provide larger databases than single-center research. This enables deeper insights into radiomics analysis and accelerates its clinical application (7, 8). However, due to variations in image acquisition protocols, processing and reconstruction settings, and imaging scanners, radiomics features exhibit batch effects (9, 10). This variability renders radiomics models non-reproducible and unstable (11). Consequently, mathematical normalization techniques that process computed radiomics feature values directly, rather than raw images, have been proposed as more practical and readily applicable approaches. The most widely used normalization technique, ComBat, has been demonstrated to eliminate batch effects in radiomics features while preserving their potential biological and pathophysiological associations (12, 13). Xu et al. (14) employed ComBat to process radiomics features extracted from multicenter PET/CT images, thereby constructing a prognostic model predicting progression-free survival in head and neck cancer. Fatania et al. (15) applied ComBat to multicenter MRI features to enhance the performance of clinically relevant radiomics risk prediction models in real-world multicenter settings. Zhou et al. (16) demonstrated that ComBat eliminates radiomics feature variability caused by different CT scanners and enhances the performance of machine learning models. However, 18% of studies employing ComBat reported no benefit following correction (12). Furthermore, most existing studies on ComBat harmonization in nasopharyngeal carcinoma (NPC) focused on CT or PET/CT imaging, and the utility of the ComBat method in MRI-based radiomics prognostic studies for locally advanced NPC (LANPC) patients across different field strengths (1.5T vs. 3.0T) and centers remains unclear. Specifically, it has not been fully validated whether ComBat harmonization can effectively mitigate scanner-related batch effects, improve the prognostic performance of OS prediction models, and enhance the consistency of risk stratification across 1.5T and 3.0T MRI datasets in LANPC.

Therefore, we proposed two main hypotheses for this study (1): ComBat harmonization can effectively reduce the non-biological variability of MRI radiomics features between 1.5T and 3.0T scanners from different manufacturers (2); ComBat harmonization can improve the generalization performance and risk stratification consistency of the radiomics-clinical prognostic model for OS in LANPC patients across multi-center datasets. This study first evaluated the impact of the ComBat harmonization method in reducing variability of radiomics features across different MRI scanners, and subsequently analyzed the effect of ComBat harmonization on the performance of NPC prognostic models, aiming to provide novel insights for multicenter radiomics research and clinical applications.

## 2 Materials and methods

### 2.1 Patients

The institutional review board approved this retrospective study and waived the requirement for written informed consent. Patients diagnosed with LANPC and treated with induction chemotherapy plus concurrent chemoradiotherapy (IC+CCRT) between January 2015 and October 2020 at Hospital 1 (3.0T MRI dataset, n = 324) and Hospital 2 (1.5T MRI dataset, n = 330) were included. The Hospital 1 dataset was designated as the training cohort, whereas the Hospital 2 dataset was divided into two validation cohorts: pre-ComBat harmonization (validation cohort 1) and post-ComBat harmonization (validation cohort 2).

**Figure 1:**
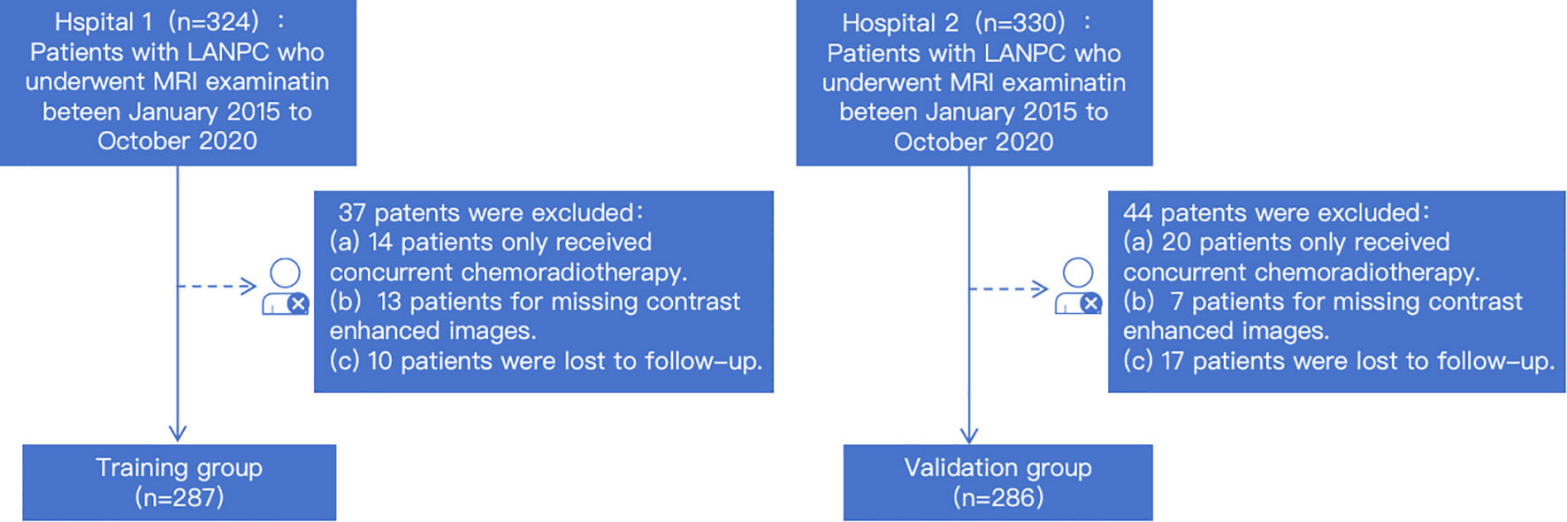
Patient selection flowchart.

The inclusion criteria were as follows (1): histopathologically confirmed NPC (2); locoregionally advanced disease (Stage II–III) according to the 9th edition of the Union for International Cancer Control/American Joint Committee on Cancer (UICC/AJCC) staging system (3); treatment-naïve status (4); receipt of IC+CCRT (5); availability of complete clinical data and high-quality MRI images; and (6) absence of other primary malignancies. The exclusion criteria included (1): presence of concurrent malignant tumors (2); unknown follow-up duration or outcome (3); incomplete MRI sequences; and (4) significant image artifacts. The patient selection process is summarized in **Figure 1**.

### 2.2 Treatment assessment and follow-up

All patients were administered IC regimens consisting of either TPF (docetaxel, cisplatin, and 5-fluorouracil) or GP (gemcitabine and cisplatin). The dosages were as follows: TPF—docetaxel 60 mg/m² and cisplatin 60 mg/m² administered intravenously every 3 weeks; 5-fluorouracil 600 mg/m²/day administered via continuous infusion over 120 hours. GP—gemcitabine 1 g/m² and cisplatin 80 mg/m² administered intravenously every 3 weeks. Three cycles were administered at 3-week intervals. CCRT consisted of cisplatin (100 mg/m² administered intravenously over 1–3 days, repeated every 21 days), which was administered for 2–3 cycles during radiotherapy.

Intensity-modulated radiotherapy (IMRT) was delivered such that the gross tumor volume (GTVnx/GTVnd) received 66–72.6 Gy (2.0–2.2 Gy per fraction), while the clinical target volume (CTV) received 56–62.7 Gy (1.7–1.94 Gy per fraction). Treatment was administered in one fraction per day, five days per week, over approximately six weeks.

Patients were followed up every 1 to 3 months during the first two years, every six months between years three and five, and annually thereafter. The primary endpoint was OS, defined as the interval from diagnosis to death from any cause or to the last follow-up for censored patients.

### 2.3 MRI acquisition

All MRI scans were acquired before the initiation of any anti-tumor treatment (IC+CCRT). The MRI acquisition protocols for both 1.5T and 3.0T scanners remained unchanged during the entire study enrollment period (January 2015 to October 2020), with no adjustments to key sequence parameters, field of view, or slice thickness.

1.5T MRI scans were acquired using a Siemens Magnetom Avanto scanner (Siemens Healthineers, Germany). The sequences included axial T_2_-weighted imaging (T_2_WI) and T_1_-weighted imaging (T_1_WI) for non-contrast scans, as well as axial, coronal, and sagittal contrast-enhanced (CE) T_1_WI, with fat suppression applied in one plane. Key imaging parameters were as follows: T_1_WI (repetition time [TR] = 450 ms; echo time [TE] = 15 ms), T_2_WI (TR = 6000 ms; TE = 95 ms), field of view (FOV) = 230 × 230 mm, matrix size = 512 × 168, flip angle = 90°, slice thickness = 5 mm, and slice gap = 0.5 mm.

3.0T MRI scans were acquired using either a Discovery MR750 (GE Healthcare, USA) or a Siemens Magnetom Skyra (Siemens Healthineers, Germany) scanner. Non-contrast scans included axial T_1_WI and T_2_WI, while contrast-enhanced scans comprised axial, coronal, and sagittal CE T1WI with fat suppression applied in one plane. The imaging parameters were as follows: T_1_WI (TR = 600 ms; TE = 11 ms), T_2_WI (TR = 3500 ms; TE = 95 ms), FOV = 230 × 230 mm, matrix size = 240 × 320, flip angle = 90°, slice thickness = 5 mm, and slice gap = 0.5 mm.

Gadolinium-diethylenetriamine pentaacetic acid (Gd-DTPA; Magnevist, Bayer Healthcare, Berlin, Germany) was intravenously administered at a dose of 0.1 mmol/kg body weight and at a rate of 2.0 ml/s for both 1.5T and 3.0T MRI examinations.

### 2.4 Image preparation and tumor segmentation

MRI data were acquired from different scanners with varying manufacturers, field strengths, and acquisition protocols, which potentially resulted in heterogeneous gray-level distributions. To mitigate these effects, images were subjected to N4 bias field correction and resampled to isotropic voxels of 1 × 1 × 1 mm³ for normalization.

**Figure 2:**
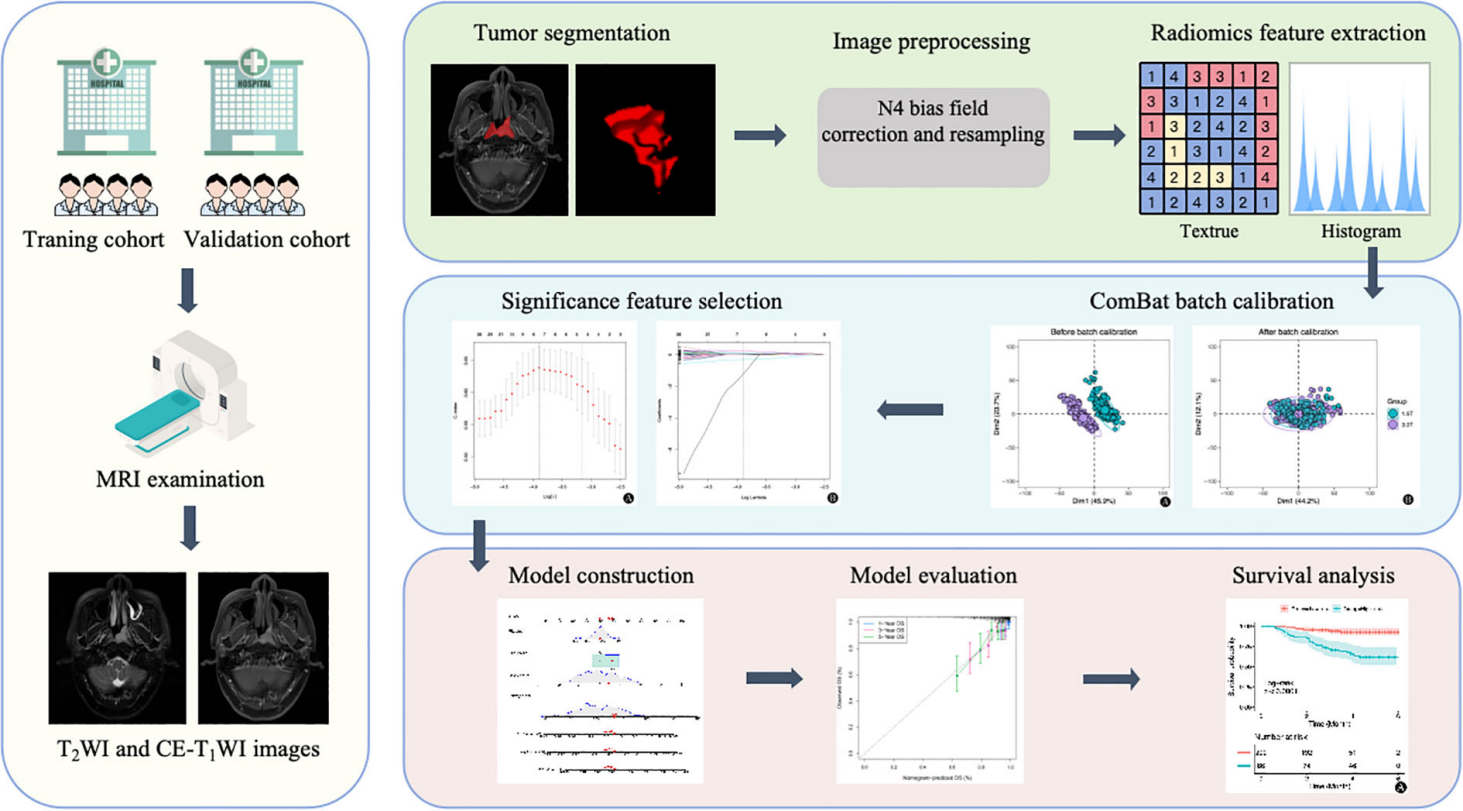
The dual-center and cross-field strength radiomics study workflow. Steps include MRI imaging, tumor segmentation, image preprocessing, feature extraction, batch calibration, feature selection, model construction, evaluation, and final survival analysis, with corresponding icons and example plots for each step.

The overall radiomics workflow is illustrated in **Figure 2**. MR images were retrieved from the Picture Archiving and Communication System (PACS). Regions of interest (ROIs) encompassing the nasopharyngeal tumors were manually delineated slice-by-slice on T2WI and CE T1WI sequences using ITK-SNAP software (version 3.8.0). The segmentations were performed by an experienced radiologist (Radiologist A, with 8 years of head and neck MRI expertise), who carefully excluded blood vessels, hemorrhage, necrosis, and cystic areas. Ambiguous cases were resolved in consultation with a senior radiologist possessing 20 years of relevant experience.

### 2.5 Radiomics feature extraction and ComBat harmonization

A total of 1,928 radiomics features were extracted from tumor ROIs on CE T1WI and T2WI sequences using the PyRadiomics software package. The extracted features included shape descriptors, first-order statistics, texture features, wavelet transformations, exponential features, and original features derived from image transformations.

**Figure 3:**
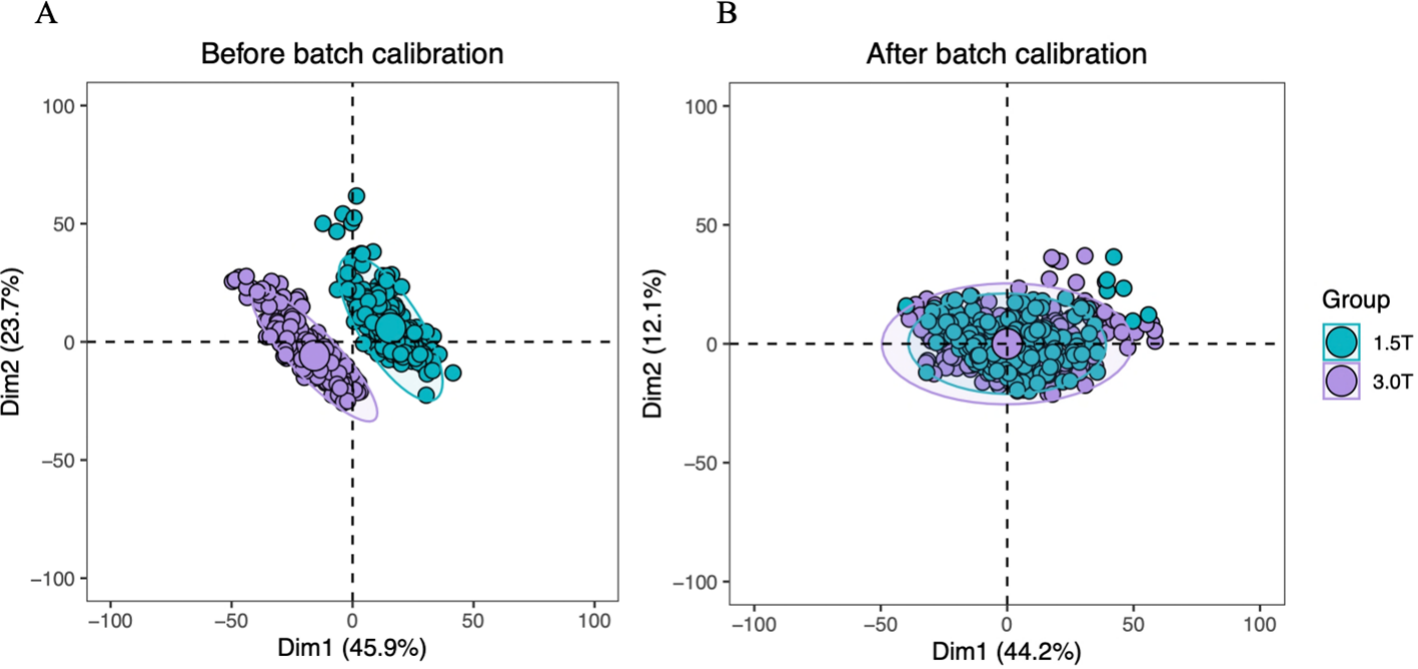
Two-panel scatter plot comparing two groups, labeled 1.5T (blue) and 3.0T (purple), before and after batch calibration. Panel **(A)** shows distinct clusters by group before calibration, while Panel **(B)** shows overlapping clusters after calibration, indicating improved group alignmen.

To eliminate inter-scanner batch effects and avoid information leakage, a reference-based ComBat harmonization approach was implemented in a strictly training-set-only manner prior to any feature selection or model development steps, in accordance with established methodological guidelines for multicenter imaging biomarker harmonization (12). The 3.0T MRI dataset from Hospital 1 was pre-designated as the fixed reference batch for ComBat harmonization, and all ComBat correction parameters (including mean and variance adjustments for batch effect normalization) were exclusively estimated using this 3.0T reference batch. No data from the 1.5T MRI dataset (Hospital 2, n=286) was involved in the parameter estimation process. This reference-based strategy was selected for multiple clinical and methodological reasons: the 3.0T scanner is the clinically predominant modality for the diagnostic workup and treatment planning of LANPC at our lead center and in most tertiary oncology institutions; 3.0T MRI provides superior spatial resolution and signal-to-noise ratio (SNR) for head and neck imaging, yielding more robust radiomic features that serve as a reliable reference for cross-scanner harmonization; and crucially, the 3.0T dataset was the training cohort for model development, and aligning the 1.5T validation dataset to this reference batch ensured strict independence between training and validation data, eliminating the risk of information leakage. After deriving the optimal correction parameters from the 3.0T reference batch, the identical ComBat model with fixed parameters was applied to harmonize the entire 1.5T MRI dataset (Validation Cohort 1) to generate the post-harmonization 1.5T dataset (Validation Cohort 2). Following ComBat harmonization, all radiomic features (training cohort, pre- and post-harmonization validation cohorts) were standardized through z-score transformation, where the mean and standard deviation for z-score were also solely calculated from the 3.0T training cohort to maintain data independence and methodological consistency. ComBat correction was performed using ComBaTool, a free online application (https://forlhac.shinyapps.io/Shiny_ComBat/), with the “reference batch” parameter manually set to the 3.0T training dataset and no survival outcomes included as covariates during harmonization to preserve biological prognostic associations (12, 13). The effect of ComBat harmonization on feature distribution was visualized via principal component analysis (PCA) (**Figure 3**).

### 2.6 Feature selection

Univariate Cox proportional hazards regression was performed on each of the 1,928 radiomic features to evaluate their prognostic significance for OS. Features with P < 0.05 were subsequently subjected to least absolute shrinkage and selection operator (LASSO) Cox regression to identify the most predictive features. Ten-fold cross-validation was employed to optimize the penalty parameter (λ) for selecting the final feature subset. The selected features were weighted by their respective regression coefficients and aggregated to compute a radiomic prognostic score (RiskScore).

### 2.7 Statistical analysis

Differences in clinical variables between the training and validation cohorts were assessed using the chi-square test or Fisher’s exact test, as appropriate. ComBat harmonization was performed using the sva package in R software. PCA was employed to visualize the distribution of features before and after ComBat harmonization. Univariate and multivariate Cox regression analyses were conducted to identify independent prognostic factors for OS, which were incorporated into a predictive nomogram developed within the training cohort and subsequently validated in independent validation cohorts.We followed key methodological recommendations by applying ComBat without including survival outcomes as covariates and performed harmonization prior to feature selection to avoid data leakage.

Model discrimination was evaluated using the concordance index (C-index). The optimal cutoff value for the nomogram-derived risk score was determined by the maximally selected rank statistics method, stratifying patients into low- and high-risk groups. Kaplan–Meier survival curves were plotted, and differences between groups were assessed using the log-rank test. Chi-square tests were used to compare risk stratification results before and after ComBat correction. Statistical analyses were conducted using R software (version 4.2.2) and SPSS (version 25.0). A two-sided P value of less than 0.05 was considered statistically significant.

## 3 Results

### 3.1 Demographic and clinical characteristics

A total of 654 potentially eligible patients with LANPC were recruited from two hospitals. After application of the exclusion criteria, 81 patients were excluded, resulting in a final cohort of 573 patients for analysis. The median follow-up time for the entire cohort was 41.9 months (range: 6–73 months). A total of 12 OS events (all-cause deaths) were recorded during follow-up. The demographic and clinical characteristics of the study population are summarized in **Table 1**.

**Table 1:** Baseline characteristics of patients in the training and validation cohorts.

Characteristics	Training cohort (n=287)	Validation cohort (n=286)	*P* value
Gender			0.769
Female	68 (23.69)	70 (24.47)	
Male	219 (76.31)	216 (75.53)	
Age (years)	45.50 (37.00, 52.00)	45.00 (35.00, 52.00)	0.404
Family history			0.456
Yes	236 (82.22)	228 (79.72)	
No	51 (17.78)	58 (20.28)	
Somking			0.144
Yes	186 (64.80)	168 (58.74)	
No	101 (35.20)	118 (41.26)	
BMI(kg/m^2^)	22.00 (20.20, 24.11)	22.48 (20.02, 24.38)	0.489
T stage^†^			0.198
T2	88 (30.66)	73 (25.52)	
T3	82 (28.57)	79 (27.62)	
T4	117 (40.77)	134 (46.86)	
N stage^†^			0.507
N1	87 (30.31)	71 (24.82)	
N2	114 (39.69)	124 (43.36)	
N3	86 (30.00)	91 (31.82)	
Overall stage^†^			0.315
III	93 (32.40)	86 (30.07)	
IVa	194 (67.60)	200 (69.93)	
WBC(×10^9^/L) ^b^	7.00 (5.88, 8.52)	7.13 (6.15, 8.57)	0.433
Hb (g/L) ^b^	139.00 (126.00, 148.00)	138.00 (126.00, 149.00)	0.725
PLT(×10^9^/L) ^b^	278.00 (234.00, 326.00)	278.00 (235.25, 336.50)	0.783
NE(×10^9^/L) ^b^	4.42 (3.40, 5.44)	4.44 (3.53, 5.59)	0.559
LYM (×10^9^/L) ^b^	1.75 (1.41, 2.26)	1.85 (1.54, 2.26)	0.14
EBV-DNA			0.772
Positive	131 (45.64)	135 (47.20)	
Negative	156 (54.36)	151 (52.80)	
ALB	39.85 (35.70,44.00)	40.21 (35.13,45.29)	0.087
LDH (g/L) ^b^	39.70 (37.60, 41.77)	40.30 (37.62, 42.70)	0.087
PLR ^b^	152.35 (119.00, 205.55)	147.21 (116.92, 190.60)	0.149
NLR ^b^	2.40 (1.80, 3.20)	2.28 (1.83, 3.08)	0.33

Categorical data are reported as number of patients (percentage), continuous data were reported means±standard deviations or Median (Q25; 75). P values were calculated by Pearson χ^2^ test or Fisher exact test.

BMI, body mass index; WBC, white blood cell; Hb, hemoglobin; PLT, platelet; NE, neutrophil; LYM, Lymphocyte; EBV, Epstein–Barr virus; ALB, Albumin; LDH, Lactate dehydrogenase; PLR, Platelet lymphocyte ratio; NLR, neutrophil lymphocyte ratio.

†According to the eighth edition of the American Joint Committee on Cancer and the International Union Against Cancer staging system.

### 3.2 PCA visualization

**Figure 3** shows the PCA distribution of radiomic features before and after ComBat harmonization. Visually, the feature clusters of the two batches were clearly separated before ComBat, suggesting obvious batch effects. After ComBat harmonization, the two clusters became more overlapping and concentrated, indicating that ComBat effectively reduced the inter-batch variability of radiomic features.

### 3.3 Screening of radiomics features

**Figure 4:**
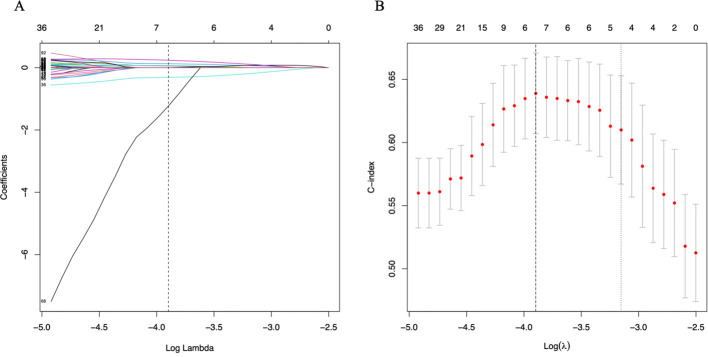
Radiomics feature selection from radiomic featrues. **(A)** Coefficient profiles of radiomics features. **(B)** The adjustment penalty parameter λ was -3.95 and 9 features were selected according to 10_fold cross validation.

A total of 205 radiomic features passed univariate Cox regression screening (P < 0.05) and were entered into the subsequent LASSO Cox regression.Before LASSO regression, all selected features were standardized using z−score transformation (mean = 0, standard deviation = 1) based on the training cohort.LASSO Cox regression was performed with 10−fold cross−validation to optimize the penalty parameter λ, using the minimum mean cross−validated error criterion. The optimal λ value was determined as −3.95, which yielded 9 non−zero coefficients corresponding to the final 9 prognostic radiomic features (**Figure 4**).

### 3.4 Construction of the radiomics signature

Nine radiomic features were identified as significantly associated with LANPC prognosis using LASSO regression with a log(λ) value of−3.95. The radiomics signature was constructed based on the following formula: RiskScore= (2.003×T1_square_glcm_Idmn)+(1.771×T1_wavelet.LHL_glrlm_LongRunHighGrayLevelEmphasis)+(0.127×T1_wavelet.HLH_glszm_ZoneEntropy)−(1.083×T1_wavelet.HHL_gldm_DependenceVariance)−(1.976×T1_wavelet.LLL_gldm_DependenceVariance)+(0.746×T2_original_shape_MajorAxisLength)+(0.696×T2_original_shape_Maximum2DDiameterSlice)+(2.089×T2_wavelet.HLH_glszm_LargeAreaLowGrayLevelEmphasis)+(3.077 ×T2_wavelet.HHH_gldm_DependenceVariance).

### 3.5 Clinical characteristics associated with prognosis

Univariate and multivariate Cox proportional hazards regression analyses identified platelet count [hazard ratio (HR), 0.997; 95% confidence interval (CI), 0.993–0.999] and Epstein-Barr virus DNA (EBV-DNA) load [HR, 2.625; 95% CI, 1.543–4.466] as independent clinical prognostic factors (**Table 2**).

**Table 2:** Univariate and multivariate cox regression analyses of clinical characteristics.

	Uni_HR	Uni_95%CI	Uni_P	Mul_HR	Mul_95%CI	Mul_*P*
Gender	1.457	1.457~0.778	0.240	–	–	–
Age	1.024	1.024~1.001	0.039	–	–	–
History	1.252	1.252~0.702	0.446	–	–	–
Smoking	1.428	1.428~0.871	0.158	–	–	–
BMI	0.983	0.983~0.910	0.652	–	–	–
WBC	1.007	1.007~0.890	0.914	–	–	–
Hb	0.993	0.993~0.979	0.379	–	–	–
PLT	0.997	0.997~0.993	0.060	0.997	0.993~0.999	0.049
NE	1.008	1.008~0.867	0.920	–	–	–
Lym	0.961	0.961~0.672	0.826	–	–	–
EBV-DNA	2.595	2.595~1.526	<0.001	2.625	1.543~4.466	<0.001
ALB	1.013	1.013~0.958	0.641	–	–	–
LDH	1.001	1.001~0.999	0.335	–	–	–
PLR	0.997	0.997~0.993	0.170	–	–	–
NLR	1.011	1.011~0.827	0.912	–	–	–

BMI, body mass index; WBC, white blood cell; Hb, hemoglobin; PLT, platelet; NE, neutrophil; LYM, Lymphocyte; EBV, Epstein–Barr Virus; ALB, Albumin; LDH, Lactate dehydrogenase; PLR, Platelet lymphocyte ratio; NLR, neutrophil lymphocyte ratio.

### 3.6 Model development and evaluation

**Figure 5:**
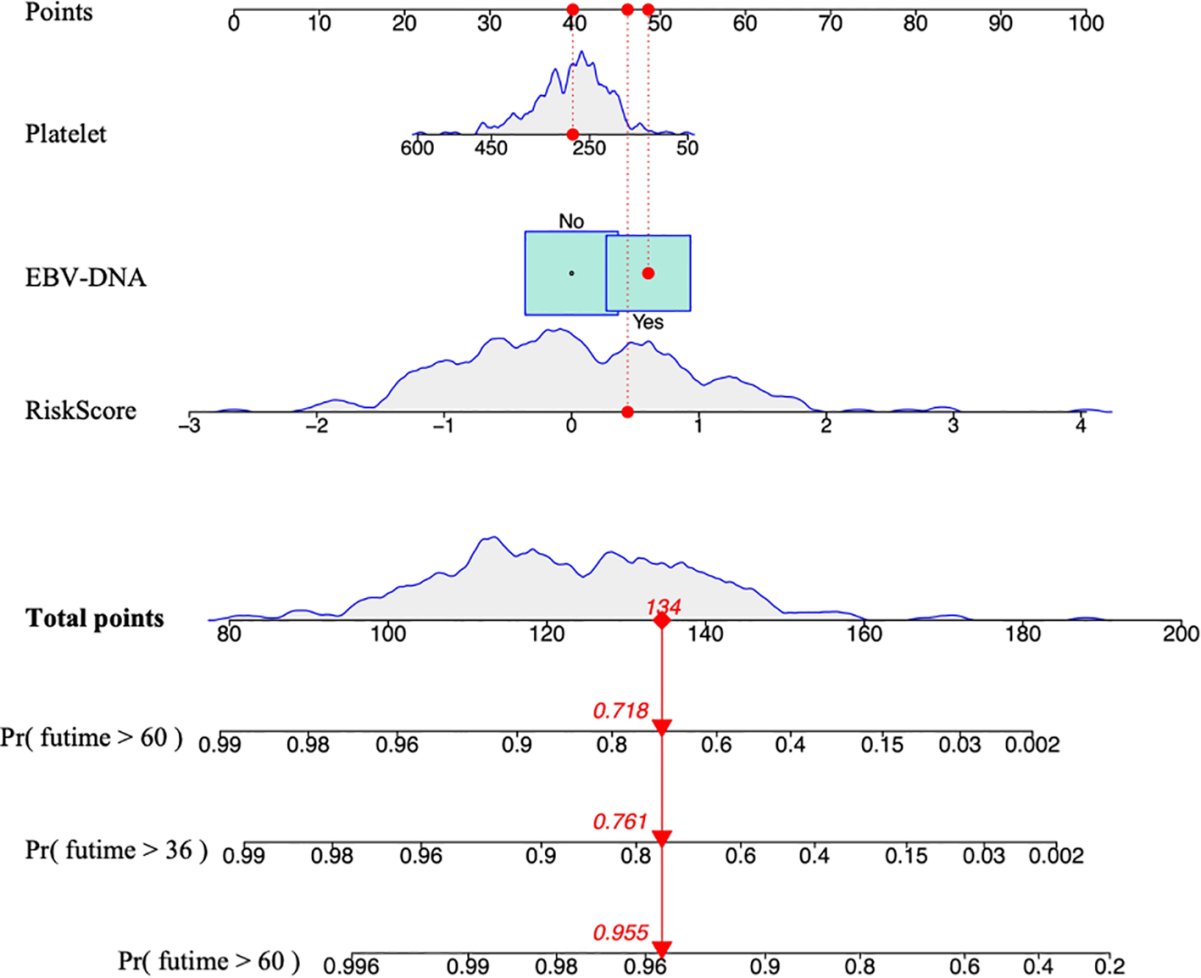
Nomogram for predicting OS in LANPC patients. Points are assigned for each prognostic factor (Platelet, EBV-DNA), and total points correspond to predicted survival probabilities. OS, overall survival; LANPC, locally advanced nasopharyngeal carcinoma; EBV-DNA, Epstein-Barr virus DNA.

**Figure 6:**
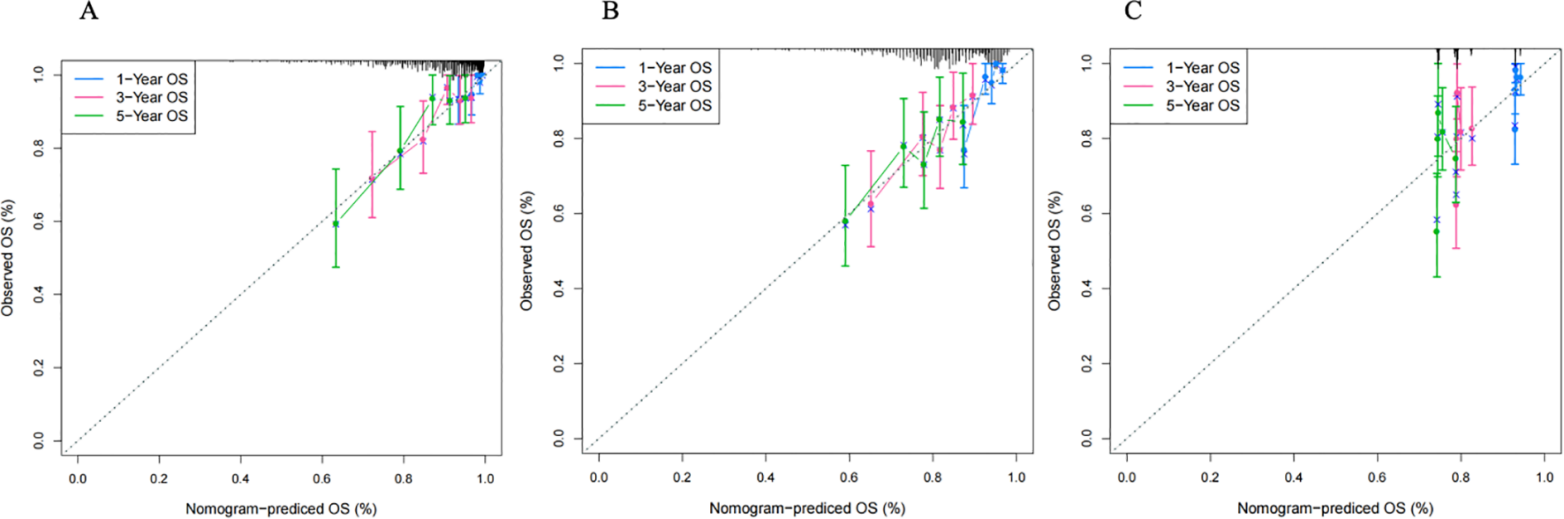
Calibration curves of the model in the training **(A)**, validation cohort1 **(B)** and validation cohort2 **(C)**. The X-axis represents the predicted OS rate. The Y-axis represents the actual OS rate. The diagonal dotted line represents a perfect prediction by an ideal model. The blue, pink, and green solid lines indicate the ability of the combined model to predict OS over 1, 3, and 5 years, respectively. OS, overall survival.

The final clinical-radiomics prognostic model was established using multivariate Cox regression in the training cohort. The radiomics risk score was incorporated as a continuous variable, together with the two independent clinical prognostic factors (platelet count and EBV-DNA load), to construct a combined nomogram for predicting OS (**Figure 5**). The C-index for OS prediction was 0.797 in the training cohort, 0.610 in validation cohort 1, and 0.648 in validation cohort 2 (**Table 3**). Calibration curves demonstrated good agreement between predicted and observed survival probabilities in both training and validation cohorts, indicating high model reliability (**Figure 6**).

**Table 3:** Performance evaluation of the predictive model.

Group	C-index	95%CI
Training cohort	0.797	0.742~0.852
Validation cohort 1	0.610	0.535~0.685
Validation cohort 2	0.648	0.576~0.720

### 3.7 Prognostic evaluation of the model

**Figure 7:**
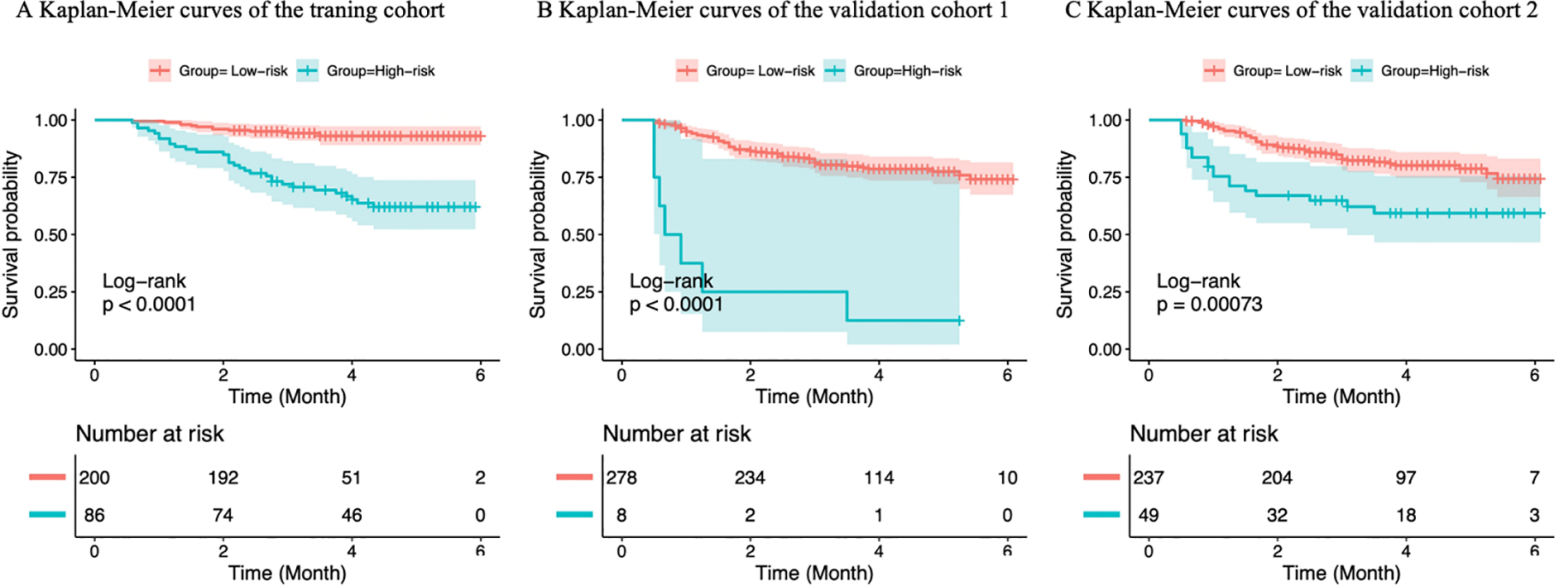
Kaplan-Meier survival curves of the model were performed to estimate OS in the training **(A)**, validation cohort1 **(B)** and validation cohort2 **(C)**. The blue and red curves represent the OS of the low- and high-risk groups, respectively. P values were calculated using a two-sided log-rank test. HR, hazard ratio; OS, overall survival; ROC, receiver operating characteristic.

Kaplan–Meier survival analysis (a metric for risk-group separation) revealed that patients classified as low-risk by the prediction model exhibited significantly better OS compared to high-risk patients across the training cohort and both validation cohorts (log-rank test, P<0.05; HR, 1.803–4.098), confirming the model’s robust ability to distinguish prognostic risk groups regardless of ComBat harmonization. Validation cohort 1 (pre-ComBat, a metric for qualitative consistency of risk stratification) identified fewer low-risk patients, reflecting substantial differences in risk stratification distribution compared to the training cohort and poor inter-cohort consistency. In contrast, validation cohort 2 (post-ComBat) exhibited a modest improvement in discrimination (C-index) compared to validation cohort 1 (0.648 vs. 0.610, **Table 3**), and more importantly, the distribution of high- and low-risk groups, the trends of survival curves, and hazard ratio values in validation cohort 2 were highly consistent with those observed in the training cohort. This improved qualitative consistency of risk stratification demonstrates the core effectiveness of ComBat for batch effect correction and its potential clinical utility for multicenter prognostic application (**Figure 7**).

The chi-square test showed that among high-risk patients, the 5-year OS rate was significantly higher in validation cohort 2 post-ComBat harmonization compared to validation cohort 1 pre-correction (17.1% vs. 2.8%, P < 0.001). Similarly, among low-risk patients, the 5-year OS rate was higher in validation cohort 2 than in validation cohort 1 (97.2% vs. 82.9%, P < 0.001) (**Table 4**).

**Table 4:** Comparison of survival differences between high- and low-risk patients across cohorts before and after ComBat harmonization assessed by log-rank test.

Log-rank test	Training cohort	Validation cohort 2
Training cohort	/	/
Validation cohort 1	<0.001	<0.001
Validation cohort 2	<0.001	/

## 4 Discussion

The objective of this study was to evaluate the impact of ComBat harmonization on the performance of prognostic models based on imaging features in a bicenter cohort of patients with LANPC. The results demonstrated that ComBat harmonization effectively corrected batch effects arising from different institutions and MR field strengths, thereby enhancing the prognostic performance of models for LANPC patients.

This study is significant because radiomics has demonstrated potential for personalizing patient treatment using routinely acquired clinical imaging data. This is particularly crucial in the clinical management of LANPC, as biological heterogeneity within a tumor—which characterizes inter-patient differences—is equally important as heterogeneity or variability observed in determining clinical outcomes (4). MRI was selected because it is an indispensable modality in LANPC management, and all patients undergoing radiation therapy will have MRI scans.

Batch effects in radiomics research refer to non-biological variability arising from heterogeneity in image acquisition, processing protocols, scanner models, or operator differences (17, 18). Such variability compromises model reproducibility and clinical applicability. By eliminating these differences, feature consistency is enhanced, enabling models to focus more accurately on key information reflecting patient pathology, thereby improving diagnostic accuracy and robustness (19). This study confirms this hypothesis: PCA validated the effectiveness of the ComBat harmonization method in mitigating device-related variability (**Figure 3**). Findings from Shiri et al. (20) and Jia et al. (21) support this outcome, demonstrating that the ComBat approach effectively eliminates radiomic feature variability caused by differences in device manufacturers, preprocessing, and acquisition parameters.

Multivariate Cox proportional hazards regression analysis identified platelet count and EBV-DNA load as independent prognostic biomarkers for LANPC, consistent with previous literature (21, 22). Elevated platelet counts are recognized as an unfavorable prognostic feature in NPC, with a prevalence ranging from 10% to 57% in malignant tumors. Although the precise mechanism underlying tumor-associated thrombocytosis remains unclear, it is influenced by interleukin-6 (IL-6) through stimulation of tumor-associated thrombopoietin, a granulocyte-macrophage colony-stimulating factor (23). Emerging evidence (24) indicates that elevated platelet counts promote tumor metastasis by facilitating immune evasion, enhancing extravasation, and suppressing natural killer (NK) cell activity. EBV infection is closely associated with NPC pathogenesis and has been widely reported as a prognostic indicator for LANPC (22, 25). Therefore, platelet count, EBV-DNA load, and radiomic-derived risk scores were incorporated into the prognostic model. The C-index values for the nomogram were 0.797 in the training cohort, 0.610 in Validation Cohort 1 (pre−ComBat) and 0.648 in Validation Cohort 2 (post−ComBat). ComBat harmonization thus yielded a modest improvement in discrimination, suggesting that reducing batch effects can recover some predictive signal obscured by scanner variability. Nonetheless, the post−ComBat C−index remained below the training performance, indicating that other sources of heterogeneity or overfitting still limit cross−scanner generalizability.Regarding risk−group separation, Kaplan−Meier analysis confirmed that the model consistently stratified patients into low− and high−risk groups across all cohorts (log−rank P < 0.001; HR range 1.803–4.098). More notably, ComBat significantly enhanced risk−stratification consistency: after harmonization, the 5−year OS rates for high−risk (17.1% vs. 2.8%) and low−risk patients (97.2% vs. 82.9%) in the validation cohort became significantly closer to those observed in the training cohort (chi−square P < 0.001). Thus, ComBat improved the cross−center transferability of the risk stratification scheme, complementing the modest gain in discrimination.These findings align with reports that ComBat can increase feature stability and cross−site consistency, sometimes with modest discriminative gains (12, 15). The observed benefits may be attributable to a clear batch definition, training−set−only parameter estimation, and a relatively large sample size. Studies with more complex batch structures or smaller cohorts may not realize similar improvements (12).

This study has several limitations. First, NPC exhibits significant geographic variation, and the sample was restricted to two hospitals within the same region, lacking external validation across diverse populations. Second, ComBat harmonization was limited to the two most commonly used MR field strengths (1.5T and 3.0T). Third, we did not perform formal inter- or intra-observer reproducibility analysis for tumor segmentation, which may introduce variability. Future studies should include such validation. Fourth, the cohort only included 12 OS events among 573 patients, resulting in a limited events-per-variable ratio relative to the number of candidate radiomic features in the initial screening. This increases the risk of overfitting and unstable hazard ratio estimates.Additionally, heterogeneity in patients’ clinical treatment regimens may have introduced confounding factors affecting survival outcomes. Future studies should incorporate additional imaging parameters and equipment to enhance the model’s adaptability and clinical utility.Future multi-modal radiomics studies integrating MRI, CT, and PET/CT features with appropriate harmonization could further improve prognostic accuracy in NPC. Future studies should incorporate additional imaging parameters and equipment to enhance the model’s adaptability and clinical utility.

## 5 Conclusions

This dual−center study demonstrates that ComBat harmonization effectively reduces scanner−related batch effects between 1.5T and 3.0T MRI datasets and improves the cross−center consistency of risk stratification in locally advanced nasopharyngeal carcinoma. The radiomics−clinical model achieved a modest improvement in discrimination and robust risk−group separation, but these findings should be regarded as exploratory due to the risk of overfitting. Further external validation in larger, prospective cohorts is essential before any clinical application can be considered.

## Data Availability

The raw data supporting the conclusions of this article will be made available by the authors, without undue reservation.
